# Lived Experiences of Male Recreational Cyclists with Patellofemoral Pain in Al Madinah, Saudi Arabia

**DOI:** 10.3390/ijerph23020171

**Published:** 2026-01-29

**Authors:** Ameen Masoudi, Ushotanefe Useh, Nomzamo Charity Chemane, Bashir Bello, Nontembiso Magida

**Affiliations:** 1 Department of Physiotherapy, School of Health Sciences, University of KwaZulu-Natal, Durban 4041, South Africa; chemanen1@ukzn.ac.za; 2 Lifestyle Disease Research Entity, Faculty of Health Sciences, Northwest University, Mafikeng Campus, Mafikeng 2735, South Africa; ushotanefe.useh@nwu.ac.za (U.U.); bbello.pth@buk.edu.ng (B.B.); 3 Physiotherapy Department, Faculty of Allied Health Sciences, Bayero University, Kano 700241, Nigeria; 4 Department of Physiotherapy, Faculty of Healthcare Sciences, University of Pretoria, Pretoria 0002, South Africa; nontembiso.magida@up.ac.za

**Keywords:** patellofemoral pain, recreational cycling, self-management programme, participation restrictions, activity limitation

## Abstract

**Highlights:**

**Public Health Relevance—How does this work relate to a public health issue?**

- This study explores the lived experiences of male recreational cyclists with patellofemoral pain (PFP), a condition that significantly limits physical activity, a key determinant of health.
- Understanding real-world challenges faced by cyclists with PFP provides insights into barriers that hinder sustained physical activity in an active population.

**Public Health Significance—Why is this work of significance to public health?**

- The findings reveal that culturally grounded factors influence symptom management, help-seeking behaviour, and exercise participation among cyclists in Saudi Arabia, which is an understudied population.
- By capturing patient-centred perspectives, this study contributes essential qualitative evidence that can inform the design of more responsive, context-specific health and rehabilitation strategies.

**Public Health Implications—What are the key implications or messages for practitioners, policy makers and/or researchers in public health?**

- The insights generated from this study can support physiotherapists and healthcare planners in developing tailored self-management and injury prevention programmes that promote safe and sustained physical activity.
- The findings highlight gaps in awareness, early interventions, and access to appropriate physiotherapy care areas that can guide policy, practice, and future community-based health initiatives.

**Abstract:**

Background: Patellofemoral pain (PFP) is a prevalent overuse injury among recreational cyclists worldwide. Despite its ubiquity, little is known about the lived experiences of people with PFP, especially in Saudi Arabia, where healthcare and cultural factors may have a specific impact on how the condition is managed. The aim of this study was to explore the lived experiences of recreational cyclists with patellofemoral pain in Al Madinah, Saudi Arabia. Method: A qualitative, descriptive design using reflexive thematic analysis was employed. Eleven male recreational cyclists aged 28–44 years diagnosed with PFP were purposely recruited from Al Madinah Physical Therapy Centre. Female participants were excluded due to cultural constraints regarding sports participation. The participants consented to participate in the study and to be audio recorded. Data were collected through semi-structured interviews using an interview guide. The interview data were transcribed verbatim and thematically analysed using Atlas.ti, version 24. Results: The thematic analysis revealed six themes highlighting the multidimensional impact of PFP. The participants described localised mechanical impairment with rapid onset during activity and persistent symptoms lasting up to two weeks. Pain was exacerbated by eccentric loading and cycling-specific stressors, such as uphill riding, leading to significant anxiety and avoidance behaviours. To maintain activity, these cyclists employed adaptive strategies, including bike modifications and self-management. Notably, PFP imposed substantial cultural and social burdens, hindering spiritual practices, specifically Salah (prayer) postures, professional duties, and family caregiving. While the participants demonstrated resourcefulness through a hybrid of physiotherapy and independent research, pharmacological relief was viewed as a transient solution. Conclusions: Patellofemoral pain imposes significant multidimensional burdens on recreational cyclists in Al Madinah, which are exacerbated by cultural practices. Physiotherapy offers targeted interventions for pain relief, functional restoration, and participation enhancement, necessitating the need for culturally sensitive management programmes.

## 1. Introduction

Cycling recreationally, rather than participating in organised events, is becoming increasingly popular in the Kingdom of Saudi Arabia (KSA) as both a form of transportation and a leisure activity [1]. Although there are benefits, as the number of cyclists has increased, so has the number of overuse injuries [2]. Pain in the knee is the most common, and patellofemoral pain (PFP) is among the most common overuse injuries among recreational cyclists [3]. PFP affects approximately 25% of the physically active population, with a point prevalence of 13.5% and an annual prevalence of 22.7% [4]. In the KSA, PFP is reported to affect nearly 30% of recreational cyclists, while globally, it impacts up to 60% of all cyclists [2]. PFP is a common musculoskeletal ailment characterised by a gradual onset of discomfort restricted to the knee’s anterior retro-patellar and peripatellar sections [5,6]. It is also described as a broad term for pain felt anteriorly around the patella, which increases during loading conditions of the knee, such as prolonged sitting, squatting, kneeling, and stair climbing [7]. The aetiology of PFP is unknown and is thought to be complex [8]. Several PFP mechanisms and risk factors have been identified in cyclists, including muscle weakness, imbalances, malalignments, lower limb deformities, overuse, psychosocial problems, and poor positioning during cycling [9]. Among the suggested triggers for knee pain are different cycling purposes, body types (especially, being underweight), performance of other sports activities, and inexperience with cycling [2].

Conservative management of PFP in cyclists often includes hip and knee exercises to alleviate pain [3]. However, conclusive recommendations remain elusive, with some patients exhibiting delayed activation of the vastus medialis, which may contribute to the development or persistence of PFP [10]. The International Classification of Functioning, Disability, and Health (ICF) framework underpins this study, providing a comprehensive structure to document participants’ functioning, focusing on participation, body functions and structures, activities, and the influence of personal and environmental factors [6]. The ICF guides patient management based on specific health needs [11]. This framework utilises a biopsychosocial model to evaluate patients according to their limitations, impairments, and participation restrictions. This holistic approach to health and illness examines the interaction of biological, psychological, and social factors [11]. This model addresses the limitations of the traditional biomedical model, which focuses solely on biological causes of disease. The biopsychosocial model considers three interrelated factors that influence health and illness. The biological aspect encompasses genetics, physiology, and physical health conditions, including how a virus or injury impacts the body. The psychological component encompasses emotions, thoughts, behaviours, and mental health, including the impact of stress, coping mechanisms, or depression on well-being. The social dimension encompasses environmental, cultural, and religious factors, including family dynamics, socioeconomic status, and social support, all of which influence an individual’s overall health. The model suggests that these elements do not operate in isolation. For example, chronic stress can increase cortisol levels, while a lack of community support might worsen recovery [12]. It is often used in medicine, psychology, and public health to create more comprehensive treatment plans compared to those based on the traditional medical model of care [13].

Although there is a growing body of qualitative research on PFP, most existing studies have focused on general experiences of living with PFP, often in Western clinical populations, and have not specifically examined recreational physical activity contexts or cultural environments outside of those settings. For example, Smith et al. [14] conducted one of the earliest qualitative explorations of PFP, investigating how individuals experienced loss of physical function, pain-related confusion, and fear-avoidance beliefs while they awaited physiotherapy treatment in a UK clinical context. Glaviano et al. [15] used a phenomenological interview approach to explore how individuals with PFP adjusted their physical activity and daily life, identifying strategies such as pacing, identifying pain thresholds, and activity modification. These studies have significantly contributed to our understanding of the general lived experience of people with PFP, particularly regarding psychological responses (e.g., fear avoidance) and how pain influences daily functioning. However, important gaps remain that our study addresses. Firstly, prior research typically recruited individuals seeking or awaiting medical care, not participants who engage in a specific sport-related activity such as recreational cycling. The physical demands, repetitive knee loading, and activity expectations of cyclists create a unique context for understanding PFP that has not been specifically explored. Secondly, existing qualitative studies have predominantly taken place in Western healthcare or community settings, with limited attention to the experiences of individuals in Middle Eastern contexts such as Al Madinah, Saudi Arabia. Sociocultural factors, including attitudes toward pain, exercise participation, and healthcare access, may shape how PFP is experienced and interpreted in this population compared to previous studies. Furthermore, recreational cyclists often view cycling as integral to their lifestyle and self-identity. Experiences of PFP in these individuals may involve distinctive themes related to sport identity, community participation, and activity adaptation, which have not been addressed in prior qualitative work primarily focused on broad physical activity modification. Finally, while earlier studies documented general activity modification and psychological impacts, they did not examine how sport-specific adjustments (e.g., changes in cycling intensity, gear selection, and route choice) influence lived experiences of pain and ongoing participation in physical activity.

In the KSA, there is an increasing prevalence of PFP in recreational cyclists, compounded by a lack of sports medicine protocols specifically addressing PFP management [1]. Managing PFP within the ICF framework and the biopsychosocial model is highly recommended in the KSA, as these approaches enable clinicians to assess the impact of knee pain on physical function and culturally significant activities, such as daily prayers, family responsibilities, and community engagement. By addressing both biomechanical impairments and psychosocial barriers, physiotherapists can design individualised interventions that support adherence and improve the overall quality of life. While cycling has been growing in popularity, there is limited research on the experiences of cyclists living with knee pain in the KSA region, where multiple cultural and recreational activities can impact the knee joint [2]. The cultural context of the KSA encompasses certain practices and daily activities, such as squatting, kneeling, and prolonged sitting, which are integral to both daily life and religious rituals (e.g., prayer) [2]; consideration of this context is essential for caring for a person with PFP. These activities may exacerbate knee pain, especially in individuals already affected by PFP [6]. Hence, this study explored the lived experiences of recreational cyclists with PFP in the Al Madinah region of the KSA, with the aim of contributing to the development of a physiotherapy self-management programme for recreational cyclists with PFP.

## 2. Materials and Methods

This study used a qualitative, descriptive design and reflective thematic analysis to explore the lived experiences of recreational cyclists with PFP in the Al Madinah region of the KSA. This research design was adopted to gain in-depth insights into how participants experienced PFP within their daily routines and cultural environment [9]. A purposive sampling strategy was employed to identify participants who met the inclusion criteria and could provide rich, relevant, and diverse information aligned with the study objectives. Participants were recruited from Al Madinah Physical Therapy Centre, where the researchers identified eligible individuals during routine clinical visits. The recruitment included participants aged 18 years and above with at least one year of cycling experience. We excluded participants with a prior history of knee surgery. Female participants were also excluded due to cultural and social norms in the region that limit women’s engagement in public sporting activities [16].

PFP is typically diagnosed clinically through patient history and physical examination, including special tests such as Clarke’s Sign and Patellar Compression Tests. Research shows that imaging, such as MRI, is not necessary for the diagnosis of PFP in most cases, as it is primarily used to rule out other knee pathologies [4,17,18]. In this study, the diagnosis of PFP was made by a physiotherapist through a comprehensive clinical assessment, including a detailed patient history, a physical examination, and exclusion of other knee pathologies such as meniscal tears, ligament injuries (e.g., ACL or MCL tears), osteoarthritis, and patellar tendinopathy. The participants were assessed for common symptoms of PFP, such as pain around the patella during activities such as cycling, squatting, or climbing stairs. Special tests, the Patellar Compression Test and Clarke’s Sign, were used to confirm the diagnosis. All potential participants were provided with thorough information about the study’s objectives and methods, along with a comprehensive information sheet outlining their rights and responsibilities, before the commencement of data collection. A written informed consent form, which included explicit approval for interviews to be audio-recorded, was signed by the participants. Appointments for individual interviews were then scheduled at times and locations that worked for both parties.

## 3. Data Collection

To better understand the participants’ physical impairment, functional limitations, and how symptoms of patellofemoral pain affect their everyday and recreational activities, the researchers created an interview guide with open-ended questions based on the ICF framework and pertinent literature on PFP and recreational cycling [19]. See Appendix A for the detailed interview guide. Researchers with experience in qualitative research and musculoskeletal physiotherapy reviewed the interview guide to verify question clarity and establish the validity of the topic. A researcher (AM) conducted semi-structured interviews in a private and quiet room at the physiotherapy centre to ensure confidentiality. The researcher reaffirmed the participants’ rights, including the opportunity to withdraw at any time and the voluntary nature of their participation, at the start of each session. To gather detailed data, the researcher employed open-ended questions, allowing individuals to answer freely, and occasionally used probing questions. To enhance the contextual richness of the data, nonverbal cues such as gestures and facial expressions were also noted and captured. A thorough grasp of the participants’ lived experiences with PFP was confirmed when further interviews yielded no new themes or insights, indicating saturation. At this point (at the eleventh participant), data collection was stopped.

## 4. Data Analysis

The qualitative data were analyzed using Reflective Thematic Analysis (RTA), following the recursive six-phase framework established by Braun and Clarke [20]. To manage the volume of data and ensure a systematic approach, ATLAS.ti (Version 24) was utilized for data organization, coding, and visualization.

Phase 1: Familiarization

Analysis began with the verbatim transcription of the Arabic interviews. The researchers engaged in “active reading” of the transcripts while listening to the original audio recordings. Initial thoughts, striking participant quotes, and potential patterns regarding the impact of PFP on cycling were recorded using the Memo function in ATLAS.ti to maintain a transparent record of early impressions.

Phase 2: Coding

Following familiarization, the data were systematically coded. Using the Open Coding feature in ATLAS.ti 24, the researchers assigned descriptive labels to segments of text. This process was primarily inductive, allowing codes to emerge from the participants’ lived experiences rather than pre-existing frameworks. The Code Manager was used to group synonyms and refine code definitions to ensure consistency across the dataset. A systematic coding process was followed to ensure consistency, resulting in a structured coding manual. This manual, which includes code definitions and representative anchor quotes, is provided in Appendix B.

Phase 3: Generating Initial Themes

Once the entire dataset was coded, the researchers shifted from codes to broader patterns of meaning. Using the Networks tool in ATLAS.ti, the researchers visually mapped the relationships between codes. This “thematic mapping” helped identify how various codes clustered together to form candidate themes (e.g., “The Loss of Identity” or “Navigating the Healthcare Landscape in Al Madinah”).

Phases 4 & 5: Reviewing and Defining Themes

The candidate themes were reviewed against the original extracts to ensure they accurately represented the data. The Analysis Tool in ATLAS.ti was used to retrieve all quotations associated with each theme, allowing the researchers to check for internal homogeneity and external heterogeneity. Themes were then refined and named to capture the “essence” of the recreational cyclists’ experiences with PFP.

Phase 6: Producing the Report

The final phase involved selecting vivid, compelling extract examples and relating the analysis back to the research question and existing literature on PFP. While the primary analysis was conducted in the original language (Arabic) to preserve cultural nuances, the final themes and representative quotes were translated into English using a back-translation method to ensure conceptual equivalence. This process followed a four-stage protocol to ensure conceptual equivalence between the source (Arabic) and target (English) languages:

1. Forward Translation: Two members of the research team (AM & BB), fluent in both Arabic and English, independently translated the selected thematic extracts into English. They focused on capturing the “latent meaning” and emotional weight of the cyclists’ descriptions of PFP.
2. Blind Back-Translation: An independent bilingual translator, who was unaffiliated with the study and blinded to the original Arabic transcripts, translated the English versions back into Arabic.
3. Reconciliation and Comparison: The research team compared the back-translated Arabic version with the original source transcripts. Special attention was paid to regional idioms specific to the Al Madinah dialect and sports-specific terminology (e.g., descriptions of “pedal stroke” or “knee clicking”).
4. Final Polish: Where discrepancies were identified, specifically regarding the intensity of pain descriptors, the English translations were refined until the back-translation perfectly mirrored the original Arabic sentiment.

In alignment with the reflexive thematic analysis approach described by Braun and Clarke [20], the first author acknowledges their active role in the production of knowledge within this study. As a physiotherapist with an interest in musculoskeletal health, the author’s interpretations were inevitably shaped by their clinical understanding of PFP. Furthermore, their position as an insider of the Al Madinah cycling community influenced the research process. In addition, their familiarity with local cycling routes and cultural norms in Al Madinah facilitated rapport with the participants. Throughout the analysis, this author engaged in a recursive process of journaling and peer-debriefing to ensure that the themes generated reflect a collaborative interpretation of the data rather than a projection of the author’s own clinical assumptions.

## 5. Results

We employed semi-structured interviews to explore the lived experiences of recreational cyclists diagnosed with PFP in Al Madinah, Saudi Arabia. A total of 11 participants consented to participate in the study, all of whom were male recreational cyclists aged between 28 and 44 years. Table 1 shows the demographic characteristics of the participants. The interviews explored the participants’ personal experiences living with PFP, with a particular emphasis on its impact on their physical activities, psychosocial well-being, and participation in recreational cycling. Thematic analysis revealed six key themes:

1. The nature and experience of patellofemoral pain;
2. Temporal patterns of patellofemoral pain;
3. Activity-linked limitations and functional disruption;
4. Psychophysical effects and coping mechanisms;
5. Community and psychosocial constraints;
6. Knee pain management strategies.

**Table 1:** Demographic characteristics of the participants.

Duration of PFP (Months)	Cycling Experience, Years	Age (Years)	Participant Code
7	7	44	001A
2	4	37	002A
18	15	35	003A
2	3	32	004A
5	1	29	005A
6	1	38	006A
12	12	34	007A
8	8	30	008A
6	6	28	009A
24	11	43	010A
9	9	40	011A

These themes reflect the multidimensional effects of PFP, encompassing impairment, activity limitation, and participation restriction. The findings, supported by participant narratives and illustrated in Table 2, provide a strong foundation for designing targeted physiotherapy interventions tailored to the specific needs of this population.

## 6. Theme 1: The Nature and Experience of Patellofemoral Pain

This theme captures the subjective experience of pain as a central impairment, primarily affecting the patella region, which impacts daily activities and cycling. The majority of participants reported pain on top, under, or behind the patella, with some reporting radiation to nearby knee regions. This anatomical distribution of pain was remarkably consistent among the participants. The participants could pinpoint certain anatomical areas of pain, demonstrating the recurrent topic of pain localisation. Numerous participants complained of soreness over the patella, as one participant clarified, *“I feel the pain exactly on top of the patella of the knee”* (A007). Others felt more intense pain in the patellar area, as described by one participant: “*The pain is under the patella exactly from inside”* (A009). Some participants reported varying degrees of pain in the patellar region, which suggested that discomfort may spread to other parts of the patellofemoral joint depending on the activity: *“The pain in the same patella may be on top of the patella or sometimes in the patella itself”* (A011). The participants described the pain associated with patellofemoral dysfunction as predominantly sharp and persistent, significantly impacting their perceived physical well-being.

The mechanical components of pain were particularly noticeable as individuals described feeling *“Like bones are rubbing against each other”* (A001) and other sensations indicating joint dysfunction. In addition to emphasising the participants’ reported level of discomfort, these descriptions also revealed their intuitive comprehension of the underlying mechanical nature of their ailment. According to the participant accounts, pain affected immediate sensations and long-term functional ability, demonstrating the progressive nature of symptoms: *“In the beginning, the movements of the same patella grow restricted and eventually halt completely”* (A005). This pattern of progression demonstrates how pain progresses from discomfort to functional limitation, indicating that PFP is a complex syndrome that affects joint mobility and overall knee function rather than just being a pain problem.

## 7. Theme 2: Temporal Patterns of Patellofemoral Pain

The participants consistently described distinct temporal patterns associated with the onset and progression of patellofemoral pain. For many, symptoms began within 3 to 10 min of cycling, suggesting a rapid response to joint loading. One participant remarked, *“Pain started after five minutes of riding the bike”* (A010), while another reported, *“I didn’t pay attention to the time in general, but we can say after five or 10 min of riding”* (A009). A few participants reported that the pain starts immediately upon initiating movement, as described by one participant, *“The pain normally starts when I start riding the bicycle”* (A005), indicating heightened joint sensitivity at the onset of physical activity. Some individuals experienced temporary relief through movement or self-initiated techniques. For instance, one participant reported, *“As soon as I rest my legs by stretching and bending, then the patella movement returns to normal”* (A005), suggesting that dynamic flexibility exercises may provide short-term comfort. However, such strategies often failed to produce lasting effects. The pain usually persisted beyond the activity itself, with some participants reporting durations ranging from 12 h to as long as one or even two weeks. For instance, one participant stated, “*Knee pain affects me especially at night; the pain can last up to 12 h*” (A001), while another noted, “*The pain sometimes stays with me for a week or even more*” (A007), reflecting the chronic and fluctuating nature of PFP. These varied experiences illustrate how the unpredictable timing and persistence of pain influence daily routines, limit physical engagement, and contribute to emotional distress. The condition affects performance during cycling and interferes with recovery periods and readiness for subsequent activity.

## 8. Theme 3: Activity-Linked Limitations and Functional Disruption

Specific limitations were noted in activities requiring knee flexion under a load, such as cycling, climbing stairs, or prolonged walking, leading to lifestyle adjustments and psychosocial effects. This theme explored the impact of PFP on daily and recreational activities where participants reported pain exacerbation during functional tasks. The subthemes that emerged from this theme included postural and mobility activities, physical activity limitations, and cycling-specific activities. During postural and mobility activities, the participants stated that pain was triggered by bending the knee (“*I feel the pain in bending the knee… such as praying,*” (A001)), squatting (“*In the squat the pains are very unbearable*,” (A001)), and stair climbing (“*There is severe friction in the patella… if there are many floors*” (A007)). The participants experienced significant restrictions across a broader spectrum of physical activities, demonstrating the impact of patellofemoral pain syndrome on their overall physical activity. These findings showed that a variety of weight-bearing and high-impact activities frequently caused pain exacerbation, resulting in substantial changes to the participants’ exercise routines and everyday physical activities. The most common causes of severe pain were found to be high-impact activities, especially those involving jumping and landing techniques. To highlight the unique biomechanical challenge of eccentric loading during landing phases, one participant reported that *“The most pain comes during jumping down”* (A005), suggesting the diminished ability to absorb shocks in the patellofemoral joint. Weight-bearing exercises, especially those that include downward movement while carrying extra weight, were also discovered to be primary pain triggers. One participant described the intensity of symptoms during loaded activities as follows: *“I feel severe pain when I go down while carrying weights”* (A004). This pattern suggests that the combination of gravitational forces and additional loading places intolerable stress on the patellofemoral joint, causing individuals to avoid such activities altogether.

The overall impact of these restrictions on behaviour was significant, with many participants stating that they had entirely given up sports and physical activities that they had previously enjoyed. These participants’ exercise choices were severely limited, and their general health and well-being might have been negatively affected by the inability to run, jog, and jump. In addition to physical activity limitations, the participants discovered that cycling-specific activities frequently increased their patellofemoral discomfort, demonstrating a clear link between the mechanics of cycling and the degree of pain. The study found that specific challenging cycling techniques and training activities significantly exacerbated the participants’ symptoms, producing recognisable triggers that affected their cycling habits and performance. Riding uphill became a primary source of discomfort, with the participants reporting a significant increase in pain during this activity. The biomechanical stresses that uphill biking places on the patellofemoral joint are highlighted in one participant’s description: *“I try to avoid the hills or avoid the mountains”* (A007); they further reported that cycling at high speeds was a problem and they needed to change their intensity to control their symptoms. One cyclist said, *“Resistance must not be high, so the pain must not increase”* (A008), showing that they understood the link between cycling intensity and pain exacerbation. In addition to the actual cycling, the participants reported greater discomfort during extra training activities, especially those that involved similar biomechanical demands: “*When I do lunge exercise, the pain becomes more*” (A006), indicating an impact from the strength and conditioning workouts that cyclists frequently perform. These results demonstrate how posture, movement, and cycling-related activities exacerbate pain, providing a clear target for biomechanical and activity-specific physiotherapy interventions. Identifying these specific triggers helps create targeted therapy strategies that address the unique needs of recreational cycling while also managing PFP.

## 9. Theme 4: Psychophysical Effects and Coping Mechanisms of Knee Pain

The profound psychological and physical repercussions of PFP on those who suffer from it require the development of a wide range of adaptive coping mechanisms to deal with the intricate interaction between emotional distress and physical restrictions. This study examined how individuals cope with the immediate physical challenges and the longer-term psychological consequences of persistent knee pain. The psychological strain of PFP was evident in the generalised fear and anxiety that spread outside of cycling-related activities. The participants shared how the unpredictable nature of their pain caused them ongoing anxiety: *“The pain makes me constantly afraid, and I cannot get comfortable”* (A004). Their quality of life was negatively affected by this persistent anxiety, which led to a cycle in which their dread of pain worsened their general distress. The participants also exhibited considerable avoidance behaviours as a result of the illness, as they began to avoid activities they had previously enjoyed: *“Sometimes I stop the exercising that I am doing due to this pain”* (A006). Another participant mentioned avoidance behaviour towards running activities: *“As for running, sometimes when I start running, the pain will definitely come”* (A005), which resulted in a decrease in overall physical activity levels. The fact that people expressed such intense emotional anguish about losing access to their favourite recreational activities was probably the most poignant aspect. One cyclist’s thoughts clearly demonstrate the deep feeling of loss: *“I get agitated, to be honest, because of this pain”* (A011). This statement captures the profound emotional effect when a favourite pastime becomes a source of suffering and restriction.

## 10. Sub-Theme 4.1. Coping Mechanisms

The participants overcame these difficulties by using a variety of adaptive coping mechanisms, demonstrating remarkable resilience. Since some cyclists discovered that pushing through early discomfort could bring relief, persistence became a prevalent strategy; as described by one participant, *“When I continue to ride… the pain will go away”* (A001). One participant discovered that some movement patterns can help control symptoms while engaging in physical activity. Some participants also learned to manage pain by making practical adjustments to their bikes. For example, biomechanical adjustments were found to ease symptoms and allow for ongoing engagement: *“If I raised the seat high a little bit, then I would feel the pain become less”* (A003). The foundation of the majority of participants’ coping strategies was conservative self-management methods. Stretching exercises were frequently used: *“ I do stretch exercises for the knee”* (A008). Some participants strategically integrated rest periods into activities: *“I try to rest for a little bit so the pain could be better, then I would carry on”* (A003). Cold therapy (applying ice) was also often used as immediate pain relief: *“Actually, I put ice after the pain”* (A011). The participants’ active involvement in managing their impairment and limitations is reflected in these diverse coping mechanisms, which show their commitment to continuing to participate in activities despite their difficulties.

## 11. Theme 5: Community and Psychosocial Constraints

In addition to the physical pain, PFP caused significant obstacles to the participants’ involvement in social and community activities that are essential to their daily lives and cultural identity. The pervasive nature of the illness pervaded many areas of the participants’ lives, including family duties, professional duties, and deeply personal religious observances.

## 12. Sub-Theme 5.1. Pain During Activities of Daily Living

The impact on regular, everyday life was especially severe, affecting even the most fundamental and culturally significant aspects of life. Religious practice, which is essential in Islamic life, turned into a considerable challenge for those who followed it. As one participant put it, the physical demands of prayer postures caused substantial pain: “*As for Salah (praying), there are always problems, especially between the two prostrations; in the sitting, I feel a strange pain while bending the knee”* (A004). This impediment to spiritual practice may cause devout Muslims physical and emotional suffering. Similarly, transportation tasks, which are vital for mobility and independence, were hampered. For many people, driving, which requires maintaining a continuous knee posture and applying intermittent pressure, has become challenging: *“I had a severe pain that sometimes made me stop driving*” (A001). This restriction had a direct impact on the participants’ ability to carry out their daily tasks and maintain their independence. Basic postural behaviours that are essential to Middle Eastern society, such as squatting, became sources of intense discomfort.

## 13. Sub-Theme 5.2. Psychosocial Life

Mental health and social bonds were impacted by the psychological strain of living with persistent pain, which resulted in a complicated network of emotional difficulties. Although some participants tried to modulate their emotional suffering, their comments hinted at underlying anxieties regarding their capacity to carry out crucial family duties: *“Truly, I am not depressed but I need to solve this problem so I can help my daddy in my best way”* (A006).

## 14. Sub-Theme 5.3. Work and Professional Life

The participants found it more and more challenging to balance workplace demands with pain management, which made their professional duties more difficult. Jobs that demanded particular motions or positions were especially challenging. Stair climbing is a common workplace requirement, with one participant observing that, *“Going up the stairs affects me during work”* (A002). This restriction compelled participants to seek out alternative solutions and accommodations. Workplace modifications became essential survival strategies, but they also brought attention to the degree of functional impairment: *“I prefer using the lift”* (A004). The preference for elevators over stairs was now a necessity rather than a convenience. While these adjustments were helpful, they continually reminded them of the limitations imposed by their illness and had the potential to impact their career prospects and professional image. Although social withdrawal eventually reduced quality of life and social interactions, it became a popular way to cope with the situation. One participant described how the fear of increasing pain caused them to avoid social and job-related activities: *“I experience pain that prevents me from going to work”* (A008). This avoidance behaviour produced a vicious circle where pain led to isolation, which could make psychological and physical symptoms worse.

## 15. Sub-Theme 5.4. Family Duties

The effect of PFP on family relationships and caregiving duties may have been the largest impact emotionally. Due to their decreased ability to participate in active childcare and family support, the participants struggled, as evidenced by comments such as, *“I feel pain in the knee when I play with the kids”* (A003). The simple pleasure of playing with children became a source of suffering and limitations. These limitations had an impact on both physical interactions and emotional connections, as well as the amount of quality time spent with family members. Additional emotional discomfort resulted from the inability to react appropriately to family needs. Actions such as rushing to a child’s aid became sources of anguish rather than displays of affection and concern: *“I feel knee discomfort when my small boy comes and I rush to him and I go down and remove him and stop”* (A011). These restrictions made people feel inadequate and reliant on others to carry out conventional family responsibilities, which increased the psychological burden of the physical challenges associated with PFP. These restrictions were all-encompassing, showing that PFP goes beyond mere physical pain and causes a cascade of limitations that impact every aspect of the participants’ social, professional, and family lives, ultimately making them question their sense of self and purpose in their community.

## 16. Theme 6: Knee Pain Management Strategies

The participants demonstrated exceptional resourcefulness in developing comprehensive management strategies for PFP, utilising a wide range of approaches ranging from self-directed interventions to formal healthcare treatments. Their management approaches reflected both cultural preferences for self-sufficiency and practical adjustments due to the limitations of available healthcare resources.

### 16.1. Sub-Theme 6.1. Self-Management of Painful Activities

The participants’ self-management strategies were primarily based on exercise-related therapies, with many learning the therapeutic value of specific physical activities. Stretching activities were especially appreciated for their accessibility and quick relief from pain: *“I do stretch exercises before praying or before any sports”* (A001). This combination of religious and recreational pursuits with therapeutic exercises shows how the participants purposefully include pain management into their daily routines. Strength training, often performed in a gym, is beneficial for long-term health and well-being. One participant acknowledged the helpful role exercise equipment has in their recuperation: “*Of course, the equipment in the gym assists this aspect”* (A011). This acknowledgement of the value of strengthening exercises shows that these participants recognise the significance of addressing underlying muscle weakness, which could be a contributing factor to their pain. Immediate symptom reduction and sustained participation in activities were made possible by supportive devices. Knee bandages have become self-management aids as they provide both psychological and physical assistance: *“Sometimes I wear a bandage, I would feel the discomfort less since the bandage serves as a support to the knee”* (A008).

Under the right circumstances, walking can be used as a form of exercise and pain relief. As one person put it, *“If I am walking in a levelled location or there is no resistance in it, pain gets a bit better”* (A009). The participants have learned to recognise the best conditions for pleasant walking: “*I feel more at ease when walking when my knee isn’t swollen”* (A007). This methodical approach to walking demonstrates that the participants understand the impact of their surroundings on their symptoms. Adjusting one’s posture has become increasingly crucial for daily pain management. The participants have learned that sitting for extended periods worsens their symptoms and devised ways to mitigate this effect. One participant described their adaptive strategy as follows: *“The pains get less if I don’t sit for long or if I alter my seating position in the car seat”* (A003). These changes necessitated ongoing awareness and adjustments to everyday habits. A dependable method for managing acute pain is cold therapy, which is especially appreciated due to its ease of use and quick results. During pain flares, the participants reported strategically using cold compresses: *“I used the cold compresses, it helps me if the agony is too severe”* (A010). This statement’s conditional form implies that the participants have a solid grasp of the optimal times for cold therapy.

Although there were differing opinions regarding athletic shoes, footwear adjustments were another self-management strategy. Some participants struggled with the aesthetic implications, even though they recognised the potential benefits: *“I feel that if I wear sports shoes, it does have an effect sometimes”* (A003). This conflict between therapeutic benefit and individual choice shows the intricate decision-making process involved in managing chronic pain.

### 16.2. Sub-Theme 6.2. Treatment and Therapy

Professional physiotherapy interventions were highly valued by the participants who had access to these services. The comprehensive nature of physiotherapy programmes that incorporate multiple exercise modalities was particularly appreciated. One participant described their positive experience: “*The physiotherapists gave me some exercises, and it makes a difference*” (A005). This multifaceted approach addresses various aspects of patellofemoral dysfunction, providing the participants with structured, evidence-based interventions. Although they offer short-term relief, pharmaceutical therapies were frequently seen as inadequate long-term solutions. The participants encountered the negatives of painkillers including their transient nature and inability to treat the underlying problems: *“I took the medicines, and while the pain subsided for a while, it returned when I started work”* (A009). Many participants sought additional management techniques as a result of this cyclical pattern of relief and recurrence.

Online resources and healthcare consultations were crucial to the participants’ management strategies. The participants were able to independently conduct research and apply evidence-based treatments since online materials were easily accessible: “*I tried using ice compresses right away after going online* (A011). The combination of expert input and independent study demonstrates the participants’ dedication to identifying efficient management techniques. The variety of management strategies used by the participants illustrates the complexity of PFP as well as the ingenuity of people with chronic pain. They developed thorough management regimens that were customised to each their own needs and situation by implementing rapid symptom relief strategies, long-term rehabilitation, and lifestyle changes.

## 17. Discussion

Using the ICF model, this study performed a thorough investigation of PFP among male recreational cyclists in Al Madinah, Saudi Arabia, demonstrating its significant influence across the domains of impairment, activity limitation, and participation restriction [21]. Patellofemoral pain characteristics, functional activities that exacerbate knee pain, psychophysical effects, coping mechanisms, community and psychosocial constraints, and knee pain mediation were the five themes that emerged, shedding light on these participants’ experiences and highlighting the critical role that physiotherapy plays in managing PFP.

### 17.1. The Nature and Experience of Patellofemoral Pain

In line with its recognised pathophysiology, which is fuelled by biomechanical overload and patellar maltracking, the participants characterised PFP as a ‘sharp’, ‘burning’, or ‘stabbing’ pain that was centred around the patella, often accompanied by sensations such as heat, prickling, or crackling. These findings are consistent with previous reports that highlight PFP as a chronic musculoskeletal disorder and a persistent barrier to physical activity, particularly in activities involving repetitive knee loading, such as cycling [3]. Moreover, the rapid onset of stiffness and pain during cycling aligns with the evidence linking PFP to synovial irritation and inflammatory responses triggered by sustained joint stress [22]. PFP symptoms can be effectively reduced by physiotherapy methods that target these processes, including pain modulation techniques, such as manual therapy, therapeutic lasers, and ultrasound [23]. The application of these strategies could be crucial in reducing symptom chronicity and addressing the public health issue, especially in light of the increasing popularity of recreational cycling in Saudi Arabia.

### 17.2. Temporal Patterns of Patellofemoral Pain

The temporal nature of PFP symptoms is characterised by rapid onset during activity, persistence beyond exertion, and fluctuating recovery periods. Early onset of pain within minutes of cycling suggests heightened sensitivity to patellofemoral joint loading, consistent with reports linking PFP to mechanical overload and synovial irritation [3,22]. While temporary relief was sometimes achieved through stretching or movement, these strategies only provided short-term comfort, supporting the evidence that unsupervised self-management rarely achieves sustained benefit [24]. The persistence of symptoms for hours or even weeks reflects the chronic and relapsing nature of PFP, which has been shown to negatively impact participation in sports and daily activities [6]. These temporal patterns therefore contribute not only to physical impairment but also to emotional distress and avoidance behaviours, underscoring the need for physiotherapy approaches that incorporate both symptom management and long-term functional rehabilitation.

### 17.3. Activity-Linked Limitations and Functional Disruption

Certain activities, such as squatting, stair climbing, and cycling against resistance, were identified by the participants as the primary causes of PFP. In the literature, these exercises have been shown to increase stress on the patellofemoral joint during load-bearing movements and knee flexion [21]. Function was further limited by the culturally rooted nature of some practices, such as *Salah*, which necessitates profound knee flexion. This reflects earlier reports linking kneeling and squatting to increased patellofemoral strain and symptom aggravation [3]. High-impact activities, including jumping and landing, are also known to intensify patellofemoral joint loading, consistent with biomechanical studies demonstrating that shock absorption during these tasks generates substantial joint stress [22]. Within the cycling context, specific challenges such as uphill riding and resistance pedalling highlight the vulnerability of the joint to overuse, supporting prior evidence that suboptimal cycling biomechanics and increased resistance can significantly improve joint loading [25]. The participants’ narratives, combined with the available data, demonstrate how PFP impacts both recreational cycling and necessary daily tasks, which is consistent with other research showing a reduction in functional ability in PFP patients [22]. To address these constraints, physiotherapy management should prioritise biomechanical changes, such as bike fitting, flexibility training, and quadriceps strengthening. Culturally sensitive physiotherapy programmes may be essential for regaining function and preventing chronic disability in the Saudi environment where national campaigns are promoting physical exercise to counteract sedentary lifestyles [26].

### 17.4. Psycho-Physical Effects and Coping Mechanisms

In addition to the physical limitations imposed by PFP, the participants also reported substantial psychological consequences, including emotional distress, activity avoidance, and anxiety regarding symptom recurrence. These findings are consistent with those of previous research indicating that chronic musculoskeletal pain frequently contributes to heightened anxiety, fear-avoidant behaviours, and reductions in quality of life [27]. Such psychosocial dimensions highlight the broader impact of PFP beyond impairment, extending to participation restrictions and overall well-being. Despite these challenges, many participants demonstrated resilience through adaptive coping strategies, such as persisting with cycling, modifying equipment, and incorporating stretching exercises. These behaviours reflect self-management efforts that align with the evidence suggesting that self-directed approaches can help mitigate PFP symptoms and promote functional activity [24]. However, reliance on unsupervised strategies may yield limited long-term outcomes, particularly when professional guidance is absent. This underscores the importance of structured, physiotherapist-led interventions that can refine coping methods and prevent maladaptive behaviours. The psychological burden associated with the loss of valued activities further emphasises the need to integrate mental health support into physiotherapy care for PFP. Previous studies noted that traditional management approaches often neglect the psychological dimension of the condition despite its substantial influence on patient outcomes [6]. For the active population of Al Madinah, structured rehabilitation programmes that integrate cognitive–behavioural and physical rehabilitation strategies may enhance psychological well-being and adherence, providing a comprehensive, and contextually relevant management approach.

### 17.5. Community and Psychosocial Constraints

PFP limited the participants’ ability to engage in daily, occupational, and family roles, consistent with the ICF framework, which emphasises the contextual determinants of health [28]. The impact of functional constraints involving knee flexion is amplified in Saudi Arabia due to the religious significance of *Salah*; previous studies found that PFP symptoms are aggravated by the movements associated with prayer [24]. Beyond physical limitations, psychosocial consequences such as distress and social withdrawal have also been associated with PFP [29]. Family and community duties are highly valued in this sociocultural environment. Physiotherapy management should therefore integrate occupational adaptations, functional rehabilitation relevant to culturally meaningful activities, and workplace accommodations, which are increasingly crucial in Saudi Arabia’s evolving labour market [30].

### 17.6. Knee Pain Management Strategies

The participants reported a range of management approaches, from self-directed activities such as stretching and footwear adjustments to physiotherapy-led strengthening programmes. While these strategies reflect known benefits of exercise and physical therapy in PFP management [31], the inconsistent treatment adherence and reliance on temporary pain relief are barriers to sustained care. Limited access to musculoskeletal services in Saudi Arabia has been identified as a significant challenge [32]. Thus, service delivery could be improved through organised, readily available programmes that combine professional therapy with evidence-based self-management education from physiotherapists. Optimising physiotherapy services in Al Madinah, where the healthcare infrastructure is growing, could significantly improve outcomes for people with PFP.

### 17.7. Implications for Physiotherapy Practice

The findings of this study show the essential role of physiotherapy in the treatment of PFP. A qualitative study investigating patients’ experiences with an 8-week therapeutic exercise program for PFP examined how patients engaged with and reflected on the exercise components over time. This study demonstrated that the participants actively engaged with and responded positively to physiotherapy-led exercise interventions as part of their knee pain management [33]. Similarly, another study exploring barriers and facilitators to exercise in PFP management highlighted that patients’ beliefs about the treatment and their engagement with physiotherapy exercises were crucial to their experience and adherence. In this context, performing self-managed exercises, including those involving loading, was identified as a key factor influencing the patients’ confidence and self-management of PFP symptoms [34]. Therefore, to address the pain and disability associated with PFP, physiotherapy is vital. Physiotherapists should not only provide exercise and pain management therapies but also offer biomechanical adjustments, such as proper bicycle fittings and personalised exercise programs, which are critical for managing activity limitations. Effective treatment requires patient education, practical modifications, and psychosocial support, all of which should be culturally sensitive. Supporting adherence, recommending appropriate equipment such as supportive footwear, and emphasising safe exercise techniques are essential physiotherapeutic techniques for overcoming the challenges of inconsistent therapy and reliance on self-management.

#### Implications for Public Health

The findings of this study indicate that a shift toward a lifestyle-centric service model that reconciles the biomechanical demands of cycling with the cultural and spiritual priorities of the Al Madinah community is needed. The participants’ narratives revealed that PFP is not merely a sports-related impairment, but a profound barrier to cultural identity, specifically regarding the deep knee flexion required for Salah (prayer) and the physical demands of family caregiving. To address these participant-derived insights, community-based programming should move beyond isolated quadriceps strengthening toward functional task integration, utilising a graded exposure framework to help patients reclaim spiritual practices such as floor-based prostration. Given the high level of self-management and online research reported by these cyclists, there is a clear opportunity for digitally mediated access pathways, such as localised Arabic-language platforms that provide evidence-based load management and bike-fit education. Furthermore, the “constant fear” and kinesiophobia identified in the participants’ psychophysical responses suggest that clinical service models must move away from “mechanical” metaphors toward Pain Neuroscience Education (PNE). By replacing “bone-on-bone” descriptions with concepts of joint sensitivity and resilience, clinicians can address the psychological distress inherent in this population, ensuring that physiotherapy interventions are both biomechanically sound and culturally sensitive to the unique social fabric of Saudi Arabia.

While the lived experiences of patients with PFP have been well-documented in Western cohorts, most notably by Smith, Moffatt, Hendrick, Bateman, Selfe, Rathleff, Smith and Logan [33], who identified themes of loss, confusion, and fear avoidance, this study provides a critical contextual extension by exploring these phenomena within the Saudi Arabian social and spiritual landscape. Our findings corroborate the presence of the pain-related anxiety and functional disruption noted in global literature but extend this knowledge by identifying the unique ‘loading environment’ of Al Madinah. Specifically, while Barton et al. [35] highlight kinesiophobia as a barrier to exercise, our data reveals that in the Saudi context, this fear is deeply intertwined with the inability to perform Salah (prayer). The deep knee flexion required for prostration and sitting between prostrations represents a unique biomechanical and spiritual ‘threshold’ that has not been accounted for in Western-centric models. Thus, the novelty of this research lies in its ability to demonstrate how the ‘universal’ experience of PFP is mediated by localised religious obligations and family structures, suggesting that the biopsychosocial model must be expanded to include a spiritual dimension when applied to Middle Eastern populations.

### 17.8. Limitations and Future Directions

This study’s male-only sample limits its generalisability across the broader population of recreational cyclists. Nevertheless, the in-depth, rich narratives provide valuable insights. Future research should adopt mixed-methods or multi-method designs to provide a comprehensive understanding of physiotherapy interventions for this population. Additionally, exploring barriers to physiotherapy access in Saudi Arabia could inform strategies for improving service delivery and engagement.

Women’s experiences with PFP and physiotherapy interventions may differ due to a range of factors, including gender-specific physiological differences, social expectations, and cultural barriers. For instance, women may face unique challenges related to body image, social perceptions of physical activity, and access to specialised physiotherapy services, which could affect their participation in cycling and adherence to rehabilitation programs. Additionally, societal norms and gender roles in some regions such as the KSA may influence women’s attitudes toward seeking treatment or engaging in physical activities such as cycling, potentially leading to differences in how they manage knee pain and recovery. Future research should incorporate female or mixed-gender samples to explore these potential gender differences and gain a more holistic understanding of how physiotherapy interventions can be tailored to diverse needs. Furthermore, expanding the study to other regions, particularly those with different cultural contexts, would allow for cross-cultural comparisons and a broader perspective on the experiences of recreational cyclists with PFP. A mixed-methods approach that combines qualitative insights with burden-of-disease data could provide a more comprehensive understanding of the impact of PFP on different populations, informing strategies for improved service delivery and engagement in diverse settings.

### 17.9. Conclusions

This study emphasises how PFP, which can cause chronic impairment, activity limitations, and participation restrictions, is a significant burden for recreational cyclists in Al Madinah. Physiotherapy and self-management techniques offer promising options for overcoming these barriers and enhancing the health and quality of life of this active population group.

## Figures and Tables

**Table 2:** Comprehensive summary of themes, subthemes and verbatim quotes.

Themes	Subthemes	Verbatim Codes
The Nature and Experience of Patellofemoral Pain	Pain Location	*“I feel the pain from the tip of the patella from the top, and then it will come from outside with severe pain...” (A008)* *“I feel the pain exactly on top of the patella of the knee” (A007)* *“It always comes on top of the knee; the problem is on top of the knee in my legs” (A001)*
	Pain Quality	*“I feel like bones are rubbing against each other” (A001).* *“I am feeling like needles or pricks” (A010)* *“What I mean is that in the beginning, the same patella movements become restricted, stop completely” (A005)*
Temporal Patterns of Knee Pain	Initial Pain Response	*“I didn’t pay attention to the time in general, but we can say after 5 or 10 min.” (A008)* *“It has almost started after 5 min of riding the bike” (A0010)* *“The pain will normally start when I start riding the bicycle. It immediately begins with movement” (A005)*
	Pain Persistence and Recurrence	*“It affects me especially at night, up to 12 h, the pains will still be there.” (A001)* *“The pain sometimes lasts with me for a week or even two weeks” (A007)* *“The pain may continue for a whole night until the night of the second day” (A0011)*
Activity-Linked Limitations and Functional Disruption.	Postural and mobility activities	*“I lift the seat of the bike so that I bend the knee in full while riding the bike” (A001),* *“Usually, the pain appears in the squatting position especially in the beginning” (A005)* *“ I mean going up many floors, the pain starts to appear.” (A006)*
	Physical activities limitation	*“If the person that I have to visit him has a lot of stairs, I stop going if there is no lift by him, I stop” (A006)* *“As for work especially for me as a physiotherapist, during the manual therapy, I sometimes have to use squatting position” (A005)* *“I feel severe pain when I go down while carrying weights” (A004)*
	Cycling-specific activities	*“Most of the time it comes to me while riding the bike and the high level places while I am walking upward, I feel the pain is becoming more or increasing. But in going downwards it will be light in the levelled surfaces...” (A008)* *“If the bike’s steering became heavy, and I increased the resistance, the pain became very strong” (A009)* *“The high ground, I feel that every time it is high, there is stress” (A0011)* *“I try to avoid the hills or avoid the mountains or the things that I will strive to do more efforts in it” (A007)*
Psychophysical Effects and Coping Mechanisms of Knee Pain	Fear and Anxiety	*“I become scared of riding the bike on a daily basis; instead of riding the bike every day, I ride the bike for 3 or 4 days per week” (0010A).* *“I try my best to avoid the roads that have many hills or uphill areas to not put pressure on the knee” (005A)*
	Avoidance and Reduced Activity	*“I mean I try to avoid it so the knee doesn’t get inflamed and I put myself in trouble because of this pain story that will be with me for many weeks sometimes or a few days” (007A)* *“Sometimes I stop the exercising that I am doing due to this pain” (006A)*
	Emotional distress	*“I get very upset, to be honest, because it’s this. The only sport that I practice and love...” (A0011)* *“It made me hate riding the bike.” (A009)*
	Service limitation	*“If the person I have to visit has a lot of stairs, I stop going if there is no lift for him, I stop” (A006)* *“As for work, especially for me as a physiotherapist, during the manual therapy, I sometimes have to use a squatting position...” (A005)*
Community and Psychosocial Constraints	Pain in the Activities of Daily Living	“*As for Salah (praying), there are always problems, especially between the two prostrations; in the sitting, I feel a strange pain while bending the knee”* (A004). *“I had a severe pain that sometimes made me stop driving,*” (A001).
	Psychosocial Life	*“Truly, I am not depressed, but I need to solve this problem so I can help my daddy in my best way”* (A006).
	Work and Professional Life	*“Going up the stairs affects me during work”* (A002) *“I prefer using the lift”* (A004).
	Family Duties	*“I feel pain in the knee when I play with the kids”* (A003) *“I feel knee discomfort when my small boy comes and I rush to him and I go down and remove him and stop”* (A011).
Knee Pain Management Strategies	Self-management of pain activities	*“I do stretch exercises before praying or before any sports”* (A001). “*Of course, the equipment in the gym assists this aspect”* (A011). *“Besides ice, I do stretch exercises for the knee.” (A008)*

## Data Availability

The paper and Appendix A and Appendix B contain all the data supporting this study’s findings. Any other information will be provided upon request.

## Appendix A. Interview Guide

Lived Experiences of Male Recreational Cyclists with Patellofemoral Pain in Al Madinah, Saudi Arabia

1. Impairment: Where exactly do you feel the pain after recreational cycling?
2. Tell me about your cycle settings currently (probe about height, seat,…)
3. Activity limitation: Does cycling affect any activities of daily living? Please state them and explain how they are affected.
4. Do you know if these activities affect your participation in your everyday duties? Like work or pray, or looking after the children. (probe: explain more how they affect your work or prayer or children).
5. What strategies have you tried to manage your pain? (e.g., rest, exercises, physiotherapy, medication, alternative methods)
6. What challenges or barriers make it difficult for you to manage your condition?
7. Is there anything else you would like to share about your experience that we haven’t covered?

Other questions will come up as I probe the patients.

## Appendix B. Coding Manual for PFP in Recreational Cyclists

Theme: The Fractured Athletic Identity Focuses on the psychological shift from seeing oneself as an ‘athlete’ to a ‘patient’.

Code: Identity Loss Definition:

Expressions of feeling like a different person or losing a core part of oneself due to inability to cycle.

Anchor Quote: “Cycling was my breath... now when I see my bike in the garage, I feel like a stranger to my own life.”

Code: Social Withdrawal Definition: Avoiding group rides or cycling community events to escape the frustration of being ‘the slow one’ or being questioned about the injury. Anchor Quote: “I stopped going to the Friday morning rides in Al Madinah. I couldn’t keep up, and I hated the looks of pity from the team.”

Theme: Cultural Navigation of Pain

Focuses on how the Al Madinah regional context and social norms influence pain management.

Code: Traditional Remedies Definition:

Mention of using local or traditional methods (e.g., specific oils, cupping/Hijama) before or alongside clinical PT. Anchor Quote: “Before going to the clinic, I tried warm olive oil rubs and Hijama, as my elders suggested it for joint ‘coldness’.”

Code: Healthcare Barriers Definition:

Reports of difficulty finding sports-specific physical therapy or clinicians who understand cycling mechanics in the region. Anchor Quote: “Most doctors just told me to ‘stop cycling.’ They didn’t understand that for us, the bike is more than just a hobby.”

Theme: Biomechanical Hyper-vigilance

Focuses on the obsessive focus on bike fit and bodily sensations.

Code: Gear Obsession Definition:

Constant adjustment of saddle height, cleats, or gear ratios in an attempt to ‘fix’ the pain. Anchor Quote: “I spent hours on YouTube and adjusting my saddle by millimeters, hoping the next ride would be the one without the sting.”

Code: Body Scanning Definition: Descriptions of monitoring the knee’s ‘status’ throughout the day or during specific phases of the pedal stroke. Anchor Quote: “Every stair I climb at work is a test. I’m always checking—is it a 3/10 today or a 6/10?”
